# Experimental study of erodible bed scoured by the debris flow in the narrow-steep gully

**DOI:** 10.1038/s41598-023-41589-1

**Published:** 2023-09-09

**Authors:** Yu Wu, Jiejie Ji, Shunchao Qi, Xiekang Wang, Dong Li, Hongtao Li, Xingguo Yang, Qiang Yao

**Affiliations:** 1 State Key Laboratory of Hydraulics and Mountain River Engineering, College of Water Resource and Hydropower, Sichuan University, Chengdu, 610065 Sichuan China; 2 Sichuan Water Development Investigation, Design and Research Co., Ltd, Chengdu, 610021 Sichuan China; 3 Technology innovation center for risk prevention and mitigation of geohazard, Ministry of Natural Resources, Chengdu, 611734 Sichuan China

**Keywords:** Environmental sciences, Hydrology, Natural hazards

## Abstract

In recent years, debris flows have frequently erupted in the narrow-steep gully of the earthquake-hit Wenchuan region, displaying high flow velocities and powerful scouring abilities. However, few scouring studies in the narrow-steep gully have been conducted. A model experiment simulated the debris flow scouring process in a narrow-steep flume, in which several important physical parameters, including the debris flow density (*ρ*), flume slope (*θ*), and grain size of the sediment (*D*), were varied to investigate their influences on the erodible strength. The experimental flows were composed of 50 L of water and grains, which scoured 2.3 m of erodible bed down a steeply inclined flume. A high-speed camera photographed the scouring processes, while a 3D laser device captured the final bed shapes. The experiments show that the debris flow first collides with the sediment at the head of the gully to form a pit, which is enlarged by continuous impact; the velocity of the debris flow out of the pit is significantly reduced due to the change in flow direction, resulting in a much lesser scouring effect after the pit; and finally, the gully bed presents the shape of a pit at the entrance and a groove in the middle and rear. The critical scour slope, where the gully bed shows scouring, increases with increasing debris flow density but decreases with increasing grain size of sediment. Following scouring, the maximum scouring depth is further positively correlated with the flume slope. In narrow-steep gullies, the gully bed is extremely susceptible to scouring by debris flow with a low density, and even headward erosion appears, at which the maximum scouring depth only increased from 148.04 to 149.97 mm, but the erosion amount had a significant increase of 36.9%. The research results have an important significance for revealing the disaster-causing phenomena and mechanisms of debris flows in the narrow-steep gully.

## Introduction

The debris flow is a kind of solid–liquid two-phase flow that contains a significant portion of mud, sand, and stones in the mountainous area^1,2^, and it rushes down mountainsides and spills out onto the alluvial fan to devastate life and property of local people^3,4^. As it descends gullies, debris flow entraps numerous loose materials, resulting in a several-fold increase in its volume, thus posing a serious threat to downstream safety^5–8^.

Debris flows that erupted in the Wenchuan strong earthquake zone are mainly gully-type, and one type of typical debris flow occurring in gullies with a small catchment area, a narrow channel, and a steep longitudinal slope has attracted attention. The post-earthquake area is rich in material sources, and once it rains, it is easy for debris flows to erupt in a short time in this kind of terrain^9^. The newly formed debris flows have the characteristics of a large-scale outburst at the gully mouth, a fast flow speed, rapid deposition, and blockage of the river, which will cause great harm to the gully mouth buildings and vegetation crops. Some scholars classify debris flow with the above distinctive features as the “narrow-steep” type if the catchment area is less than 5 km^2^, the integrity coefficient of watershed morphology (the ratio of catchment area to the square of gully length) is less than 0.4, and the gully slope is greater than 0.3^10–12^. Narrow-steep gully debris flows are difficult points for debris flow warning and engineering control, which are weak links in the current debris flow research.

Gully bed erosion studies have been carried out mainly by means of model experiments, and a wealth of research results have been achieved. Most of these studies focus on clear water and cover the initiation mechanisms of clear water scouring gully sediments^13,14^. However, as two different fluids, clear water does not reflect the true scouring characteristics of the debris flow. In recent years, there has been an increase in the number of studies relating to the effects of debris flows on gully bed erosion. He et al.^15^ found that the scouring effect of debris flows was more pronounced in straight gullies than in curved gullies. Zhu et al.^16^ obtained a calculation method for the scouring depth of dilute debris flows through theoretical analysis. Pan and You^17–19^ found that the depth of debris flow scour is strongly influenced by the nature of the fluid, the longitudinal slope of the gully, and the composition of the sediment. However, most scour tests have mainly been done with a fixed slope or by varying the slope to a small extent and do not reflect the typical characteristics of the narrow-steep gully with steep longitudinal slopes and a wide range of slope variations^15–17,19–23^. Notably, Baggio et al.^24^ regarded the gully slope as the major control variable and further discovered that the correlation between slope and erosion depth becomes satisfactory only for slopes steeper than 14°, but low slopes (5.7°–12.4°) are associated with a great heterogeneity in erosion depth. Besides, Haas et al.^25^ found that there are thresholds of slope that need to be overcome before significant basal scour occurs. It is therefore necessary to carry out further systematic research into the scouring patterns of debris flows by conducting flume tests.

This paper attempts to investigate the relationship between some scouring parameters (e.g., the maximum scouring depth and scour shape) and flume slopes with a wide range, and to consider several types of debris flow density and grain size of sediment by carrying out 47 flume experiments.

## Materials and methods

### Experiment setup

**Figure 1:**
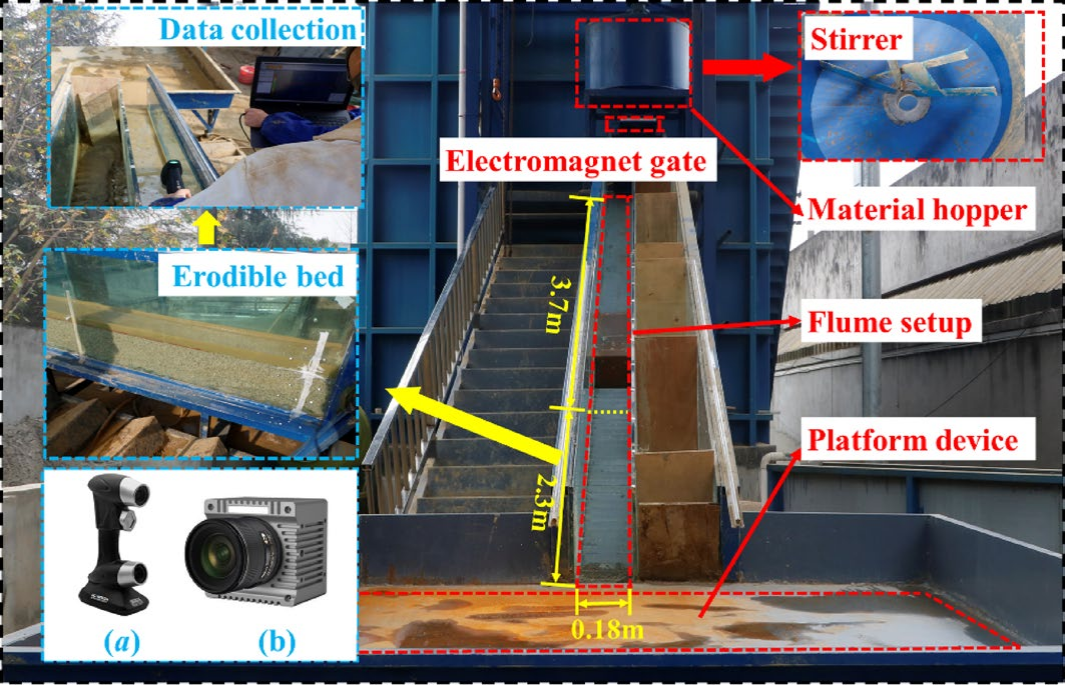
Experiment setup (**a**, Scantech Prince 335; **b**, Revealer 5F04).

In Fig. 1, an experimental model is designed to simulate the process of debris flows scouring in a narrow-steep gully, with a wide range of slopes. The experimental model consists of three parts: a material hopper, a flume setup, and a platform device. By controlling the electric hoist to raise the height of the material hopper and synchronously sliding the rollers of the flume setup and the platform device on the guide rail, the flume slope changes from 0° to 45°. The material hopper is used to store debris flows, and a stirrer is configured in it to prevent debris flows from settling. The flume setup has a length of 6 m and a width of 0.18 m, with the first 3.7 m being non-erodible, and the last 2.3 m being erodible. The platform device is utilized to collect the debris flows and bed sediment scouring out from the flume setup. At the beginning of the scouring experiment, the debris flows are released into the flume setup by opening the electromagnet gate. The debris flow passes through the non-erodible bed section to gain velocity, then flows into the erodible bed, and finally stays on the platform device.

In the experiment, a handheld 3D laser scanner, the Scantech Prince 335, is used to scan the erodible bed surface scoured by debris flow with a maximum resolution of 0.020 mm, a maximum scanning accuracy of 0.03 mm, and a scanning speed of 265,000–320,000 times per second. At the same time, the Revealer 5F04 high-speed camera is selected to monitor the debris flow motion process and the maximum resolution is 2320*1720 (4 million pixels), and the acquisition speed is 1–152,000 frames per second.

### Experiment materials

Model experiments generally need to satisfy three similarities in geometry, motion and dynamics. It is difficult to ensure that all conditions are similar due to many factors affecting the experiment. At present, there is no clear similarity law in the debris flow model test at home and abroad. Due to the limitations of the experiment theory and experiment device, the similarity conditions are mainly considered from the key indicators such as particle gradation, debris flow density, gully size, gully slope, etc.

**Figure 2:**
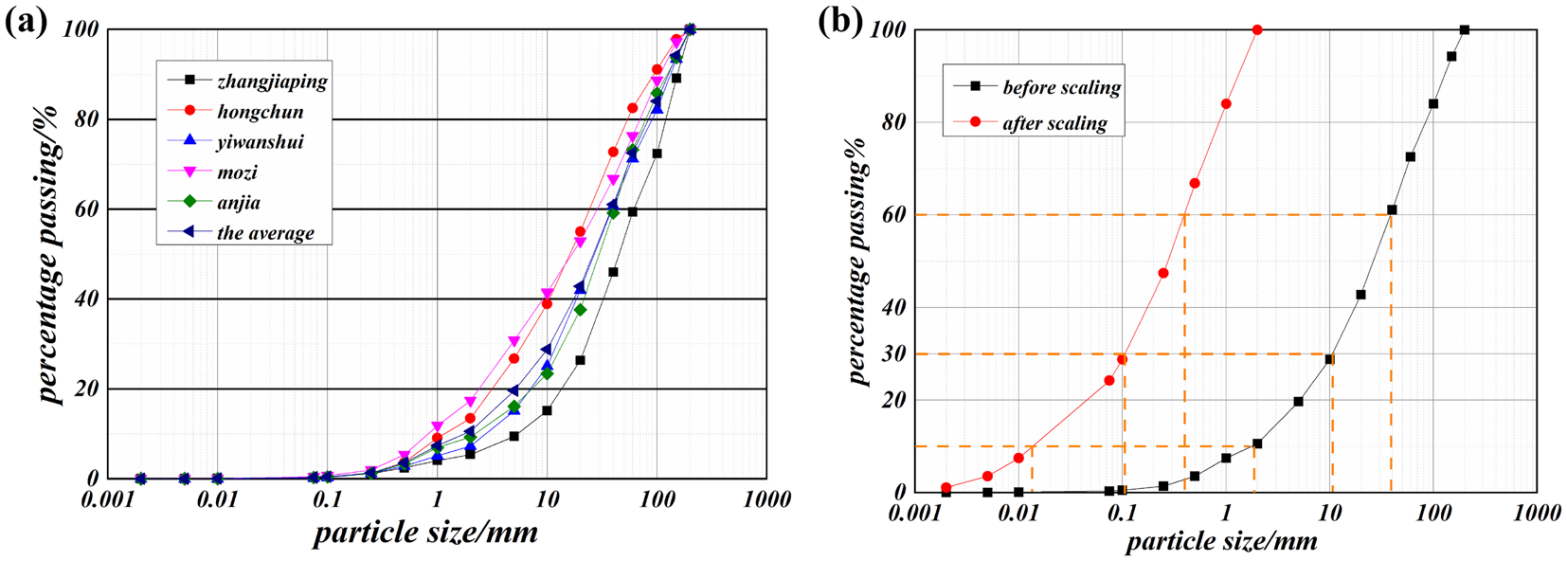
The solid composition gradation of debris flow (**a**, the solid gradation data of five typical debris flows and the average; **b**, the gradation curves before and after scaling the solid particles).

The experiment uses the mean solid particle gradation of five typical narrow-steep gully debris flows obtained from field surveys, and the maximum particle size of it is 200 mm, as shown in Fig. 2a. The debris flow in the experiment needs to have a certain degree of fluidity while ensuring that no significant solid–liquid separation occurs. It is found in the experiment that the above requirements can be met when the maximum particle size of the solid particles that make up the debris flow does not exceed 2 mm. As a result, the ratio of geometric similarity is determined to be 1:100 in this experiment. In Fig. 2b, after scaling down the gradation data 100 times, the two characteristic coefficients of the solid particle grading curve, including the uniformity coefficient and curvature coefficient, are 21.80 and 1.66, respectively. The above two characteristic coefficients are close to those before scaling, which are 21.90 and 1.60, respectively. Thus, the geometric similarity of 1:100 meets the similitude requirements of the model experiment.

The densities of the five typical debris flows are concentrated in the range of 1.60–1.90 g/cm^3^, and few parts exceed 2.00 g/cm^3^^26–28^. In this experiment, in order to truly reflect the nature of the debris flow, the debris flow density is set at a ratio of 1:1 with the field debris flow density. Therefore, the debris flow densities are set at 1.70 g/cm^3^, 1.75 g/cm^3^, and 1.80 g/cm^3^ to reflect the impact of debris flow density on erosion. The solid–liquid combinations of the debris flow at different densities used in the experiments are shown in Table 1, and information on the solid gradation is shown in Fig. 2b. Based on the debris flow used for the experiments, a Physica MCR301 rheometer is used to carry out debris flow rheology tests to obtain the coefficient of viscosity at each density.

**Table 1:** The composition of the debris flow.

Debris flow density (g/cm^3^)	Mass fraction of solid particle (%)	Mass fraction of water (%)	Viscosity coefficient (Pa.s)
1.70	68.19	31.81	0.25
1.75	70.80	29.20	0.50
1.80	73.75	26.25	0.88

Statistics show that the gully width of narrow-steep gully debris flow is mainly concentrated in 5–30 m. The flume width in this paper is designed at 0.18 m to maintain consistency in geometric similarity. According to the similar debris flow model experiments^19,25,29^, the width of the flume is within 0.09–0.3 m, implying that a flume width of 0.18 m can meet the experiment requirements. The width of the gully should be greater than 5 times the maximum grain size of the bed sediment, which should not exceed 10 mm for this experiment. The bed sediment is made up of reasonably homogeneous solid particles with grain sizes of 1–2 mm, 2–5 mm, and 5–10 mm, respectively.

In the quantitative judgment of the narrow-steep gully debris flow, the gully slope is generally greater than 0.3 (~ 16.7°). Therefore, the flume slope is designed as 10°, 13°, 16°, 19°, and 22°, where 16° can be approximated as the critical slope for narrow-steep debris flow, in order to more effectively explore the differences in scour in the steep and gentle gully.

### Experiment scheme

The movement process of debris flow scouring sediments is very complex, and its influencing factors include fluid conditions and gully bed conditions. Experimentally, debris flow density (*ρ*), flume slope (*θ*) and grain size of the bed sediment (*D*) are selected as control factors. In the experiment, the bed sediment is in a natural accumulation state, with the surface of the bed sediment level with the non-erodible section of the flume. The dense degree of the bed sediment is about 0.52, and its moisture content is about 6.2%. The volume of debris flow is 50 L. A severe scouring action appears when the debris flow density is 1.70 g/cm^3^, and the grain size of the bed sediment is 5–10 mm, so two flume slopes of 4° and 7° are added to explain the scouring action further. Finally, a total of 47 groups of scouring experiments are carried out (Table 2).

**Table 2:** Test scheme.

Flume slope		*ρ/*(g/cm^3^)		
		1.80	1.75	1.70
*D* /(mm)	1–2	10° 13° 16° 19° 22°	10° 13° 16° 19° 22°	10° 13° 16° 19° 22°
	2–5	10° 13° 16° 19° 22°	10° 13° 16° 19° 22°	10° 13° 16° 19° 22°
	5–10	10° 13° 16° 19° 22°	10° 13° 16° 19° 22°	4° 7° 10° 13° 16° 19° 22°

Note: In this paper, the experiment group is expressed by (*θ*)–(*ρ*)–(*D*). E.g., experiment group 10–1.80–1 ~ 2 represents *θ* is 10°, *ρ* is 1.80 g/cm^3^, and *D* is 1–2 mm.

The purpose of this experiment is two-fold: firstly, to observe the entire process of debris flow scouring the bed sediment, and secondly, to analyze the resulting shape of the bed after scouring. Throughout the experiment, a high-speed camera is used to record the entire scouring process on the front side of the flume, while a cell phone is set up on the side to observe the infiltration of debris flow. Additionally, a handheld 3D scanner is used to scan the erodible bed section scoured by the debris flow. The debris flow velocity is measured using the buoy method, which involves continuously throwing foam buoys into the flume and calculating the velocity based on their position at different moments. Furthermore, the debris flow depth is measured by tracing its path over the clear glass on both sides of the flume.

## Experiment results

### Scour shape

**Figure 3:**
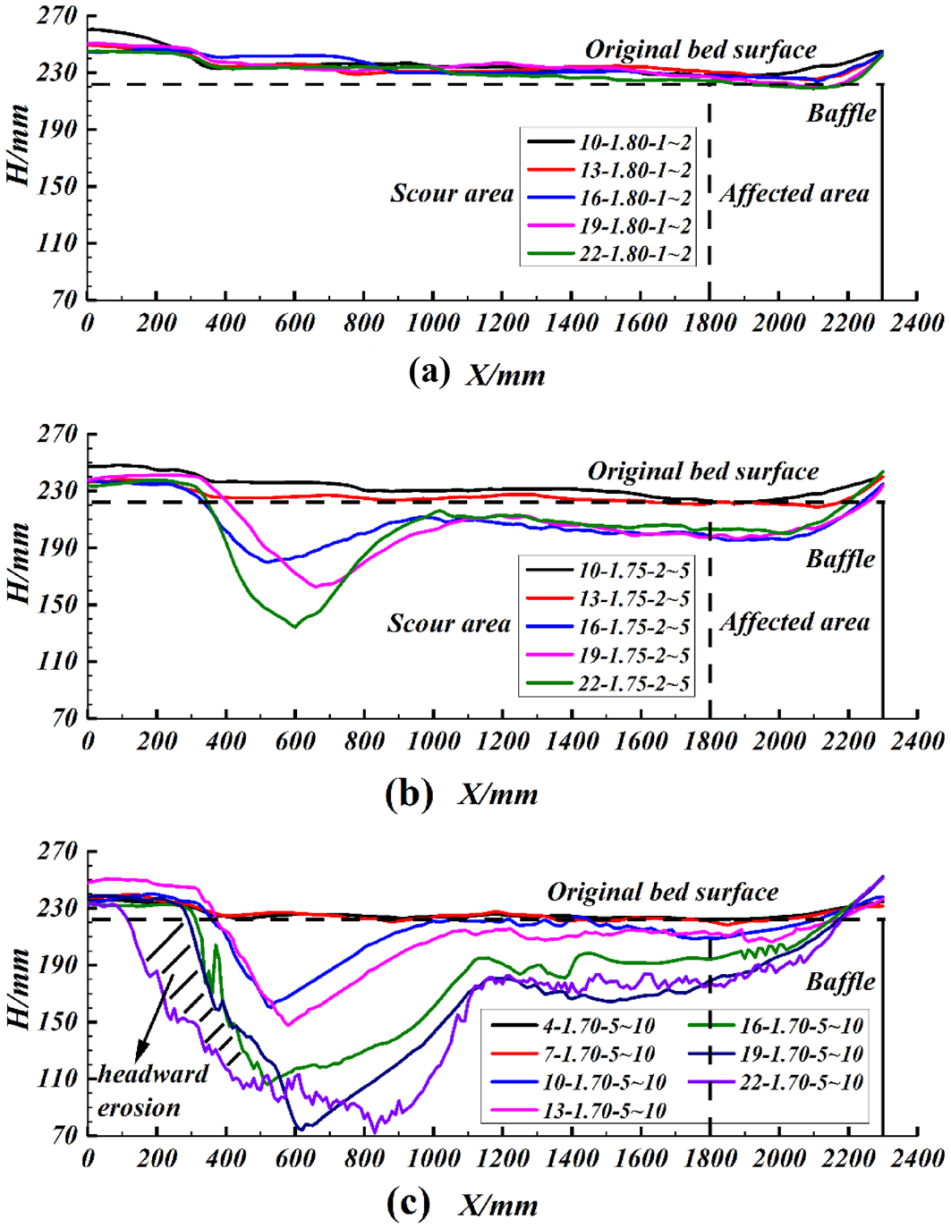
Longitudinal section of the erodible bed at the maximum scouring depth (where *H* is the sediment height; where *X* is the distance to the entrance of the erodible bed).

The erodible bed model after scouring is captured by a 3D laser scanning device. It is difficult to represent the difference in results for different experimental conditions by directly using the erodible bed model. In this paper, the longitudinal section where the lowest point is located after scouring is extracted from the 3D model as the scour shape for analysis. Figure 3 shows some shapes of the erodible bed after scouring experiments, and the rest can be found as in Supplementary Fig. S1 online. At the end of the flume, the debris flow front collides with the baffle, resulting in scour enhancement and a buildup on the baffle side. Therefore, in the scour shape analysis, only the erodible bed section within 1.8 m is focused on to avoid the influence of the baffle. The experimental results show that there are two states of deposition and scouring on the bed. In the case of deposition, its thickness decreases gradually from the head to the end of the erodible bed (Fig. 3a).

Special attention needs to be paid to scouring, and there are many noteworthy phenomena. The overall feature of the scour shape is that there is a pit at the entrance and a groove at the middle and rear, and the depth of the scouring pit is far greater than that of other positions. With the enhancement of scouring, for example, as shown in Fig. 3b and c, when the density of debris flow is 1.7 g/cm^3^ and the particle size of sediment is 5–10 mm, the slope of the trough increases from 10° to 19°, the position of the maximum scouring depth will tend to move downward, the scouring pit will continue to expand and the depth of the scouring groove will also gradually increase. When the flume slope increases to 22°, a large number of collapses have occurred in the sediments at the head of the bed, that is, the particularly severe headward erosion has occurred. It can also be found that the scouring initiation points all appear at the head of the erodible bed. Only when headward erosion occurs, the scour initiation point will move significantly forward.

**Figure 4:**
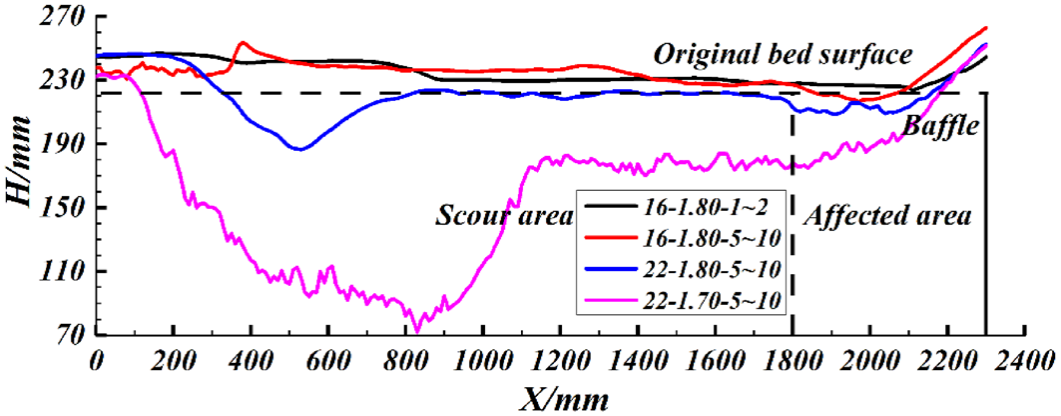
Some typical erodible bed shapes (where *H* is the sediment height; where *X* is the distance to the entrance of the erodible bed).

**Figure 5:**
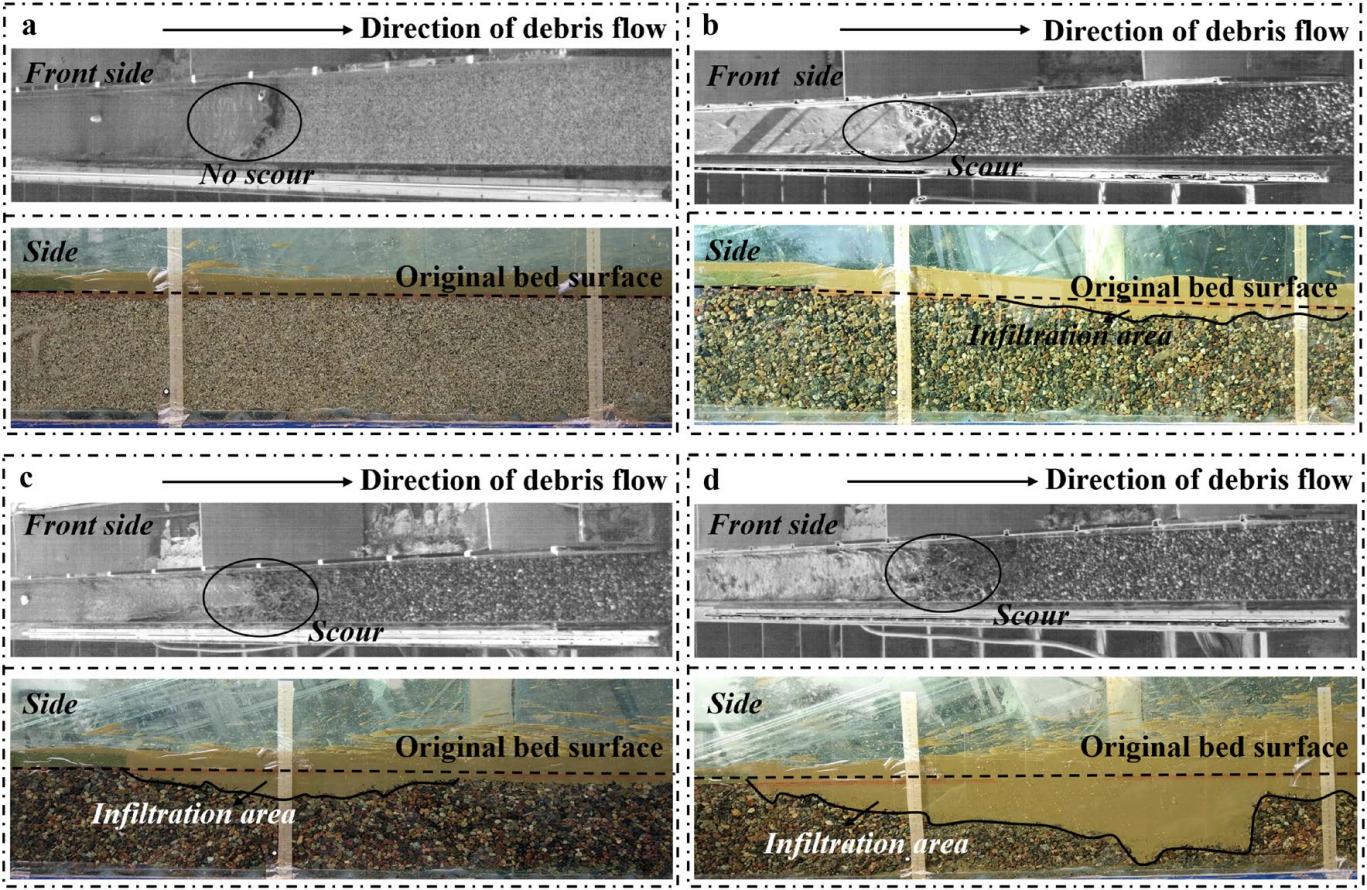
Some typical scouring processes including front side and side (**a**, experiment group 16–1.80–1 ~ 2; **b**, experiment group 16–1.80–5 ~ 10; **c**, experiment group 22–1.80–5 ~ 10; **d**, experiment group 22–1.70–5 ~ 10).

The formation process of depositing and scouring is further analyzed by the video recorded by the high-speed camera. When the debris flow density is high and the grain size of the bed sediment is small, such as the experiment group 16–1.80–1 ~ 2. As shown in Fig. 5a, at this point, the debris flow did not scour the sediment at all but flowed directly down the bed surface, and finally, part of the debris flow deposited up on the erodible bed (Fig. 4). As the grain size of the bed sediment increases, for example, experiment group 16–1.80–5 ~ 10, the contact area between the debris flow and the sediment becomes larger, resulting in an increase in the impact of the debris flow on the sediment, and thus part of the sediment at the bed head is scoured, as shown in Fig. 5b. However, due to the high debris flow density and the high viscosity between the debris flow and the bed sediment, it is difficult for the debris flow to infiltrate, resulting in more debris flow blockage in the area where the bed sediment is scoured. Despite the scouring that occurred during the experiment, the final result of the bed shape still shows full cross-section deposition (Fig. 4). When increasing the slope of the flume, like experiment group 22–1.80–5 ~ 10, as shown in Fig. 5c. The flow rate of the debris flow increases, resulting in a larger pit at the bed head. It is difficult for the debris flow to continue to fill the scoured bed sediment, so the final scour shape is shown in Fig. 4. When reducing the density of the debris flow, for instance, experiment group 22–1.70–5 ~ 10. In Fig. 5d, strong collision and scouring action occurs between the debris flow and sediment at the head of the bed, so a larger pit can be formed at the head of the bed at the beginning, resulting in a change in the direction of the debris flow velocity and continuous downward impact on the pit. Due to impacting the pit, debris flow consumes most of the energy, so the impact capacity of the debris flow behind the pit is substantially reduced. The final scour shape of a pit at the entrance and a groove in the middle and rear is formed (Fig. 4).

### Critical scour slope

For a given gully, when the debris flow and sediment conditions are determined, there is a critical slope at which debris flow neither scours nor deposits^18,19^. What must be known is the situation of neither scouring nor depositing only exists in theory. Neither reality nor experiment can satisfy the theoretical situation. Therefore, it always manifests itself as scouring or deposition on the gully bed, as shown in Fig. 3. However, through experimentation, it is possible to determine a slope at which the bed shape shows the scour. For example, in Fig. 3b, under experimental conditions with a debris flow density of 1.75 g/cm^3^ and a sediment grain size of 2 ~ 5 mm, the bed shape is full cross-sectional deposition when the flume slope is 13°, while scouring occurred at the head of the flume bed at a flume slope of 16°, so that the critical scour slope can be determined to be in the 13°–16° range. The critical scour slope for each experimental condition is obtained statistically in combination with the scour shape, as shown in Table 3. It can be found that the critical slope increases with the increase of debris flow density and decreases with the increase in grain size of the bed sediment. As analyzed by the scour shape, the higher the density of the debris flow and the smaller the sediment size, the weaker the scouring capacity of the debris flow on the sediment and the easier it is for the debris flow to fill previously the scoured sediment, which means that the gully bed is more likely to appear as a full cross-sectional siltation, resulting in a higher critical scour slope. When the debris flow density is 1.70 g/cm^3^, the critical scour slope is all less than 13°, so less dense debris flows should be given special consideration in narrow-steep gullies.

**Table 3:** Critical scour slope under different experimental conditions.

Critical scour slope		*ρ/*(g/cm^3^)		
		1.70	1.75	1.80
*D*/(mm)	1– 2	10–13	19–22	> 22
	2–5	10–13	13–16	16–19
	5–10	7–10	10–13	16–19

### The maximum scouring depth

**Figure 6:**
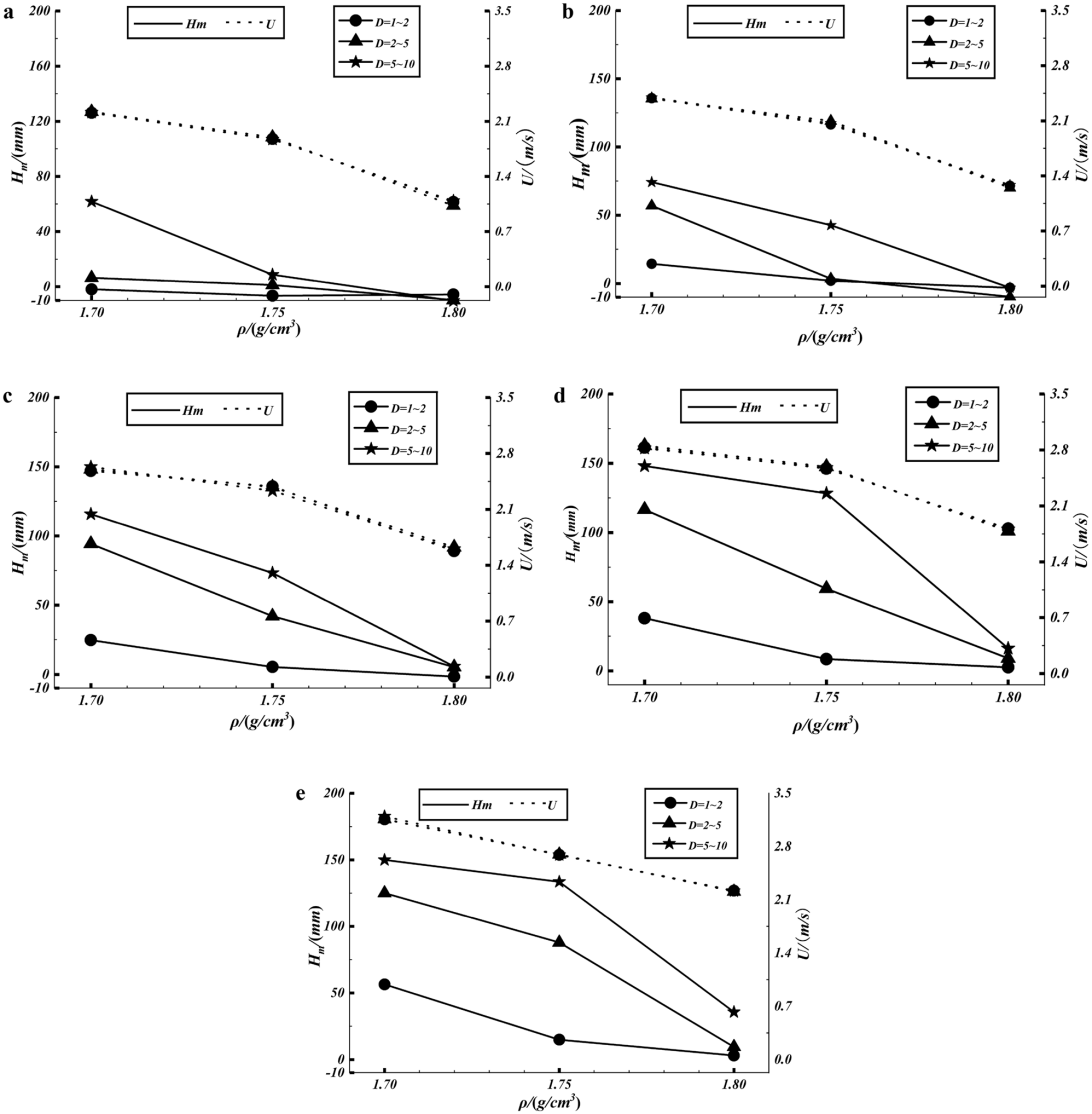
The maximum scouring depth and the velocity of the debris flow: (**a**) the flume slope is 10°; (**b**) the flume slope is 13°; (**c**) the flume slope is 16°; (**d**) the flume slope is 19°; (**e**) the flume slope is 22°.

The maximum scouring depth (*H*_*m*_) is important research data to manifest the erosion intensity of debris flow, and its value is accurately obtained based on 3D laser technology. By this method, the maximum scouring depth is calculated, while statistics the debris flow velocity (*U*), as shown in Fig. 6. It can be found that *H*_*m*_ decreases with the increase in debris flow density, showing a negative correlation. One reason for this is that when the density of a debris flow decreases, the velocity of the flow decreases accordingly, resulting in a weakening of the scouring dynamics of the debris flow. Besides, the higher the density of a debris flow, the more solids it contains. As the volume fraction of debris flow solids increases, the ability to penetrate the erodible bed decreases, which also leads to a weaker scouring capacity.

However, the contribution of the grain size of the bed sediment (*D*) to the scouring intensity is the opposite. On the one hand, as the grain size of the bed sediment increases, the pore size increases, resulting in a greater infiltration capacity of the debris flow into the gully bed, which means that the gully bed is more susceptible to scouring by debris flow. On the other hand, the increased particle size of the sediment also increases the roughness of the streambed surface, which can also lead to the gully bed being more susceptible to scouring by debris flows.

**Figure 7:**
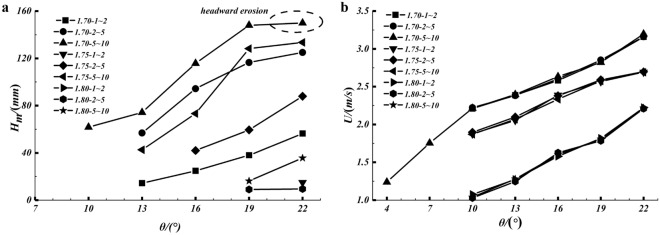
(**a**) The maximum scouring depth and flume slope; (**b**) the velocity the debris flow and flume slope.

Figure 7a shows the relationship between maximum scouring depth and flume slope, and Fig. 7b shows the relationship between debris flow velocity and flume slope. It is clear that the maximum scouring depth increases with increasing slope of the flume. In Fig. 7b, as the slope of the flume increases, the debris flow velocity increases accordingly, resulting in an increase in the scouring capacity of the debris flow. It can also be seen that the debris flow rates are essentially the same for the same debris flow density and slope of the flume, which is an indication of the stability of the experiment to some extent. In addition, the stability of riverbed sediment under large slope is lower, which leads to the formation of scouring, the sediment around the scouring pit is more likely to collapse, forming more serious scouring, even headward erosion. For example, under experimental conditions with *ρ* of 1.70 g/cm^3^ and *D* of 5–10 mm, as the flume slope adjusts from 19° to 22°, the Froude number of the debris flow increases from 5.7 (the flow velocity is 2.82 m/s, the flow depth is 25 mm) to 6.46 (the flow velocity is 3.2 m/s, the flow depth is 25 mm), and the stability of the bed sediments decreases, resulting in a large amount of collapse of the sediment around the flume and the headward erosion has occurs. Here, the maximum scouring depth only increased from 148.04 to 149.97 mm, but the erosion amount increased from 2.06 × 10^7^ to 2.82 × 10^7^ mm^3^, a significant increase of 36.9%.

As mentioned above, the larger velocity of debris flow on the steep slope is more likely to scour the gully, and the weaker stability of the riverbed sediment on the steep slope leads to the more likely collapse of the riverbed sediment, leading to a significant increase in scouring.

## Discussion

Scholars have focused for a short time on the debris flow that occurred in the narrow-steep gully^12^. These debris flows show high flow velocity and destructive force, immediately entraining many gully materials^10,11^. This type of debris flow can easily scour the gully, destroying the barrage and other control structures. Considering the potential danger of this type of debris flow, a narrow-steep flume model with a wide range of slopes was designed to research and focused on the disaster-causing phenomena and mechanisms.

For the scour shape, a scour pit is evident at the entrance of the gully in the experiment, as observed by Haas^25^ and Li^8^ in their experiments. Li attributed the increasing size of the pit to a change in the direction of the subsequent debris flow. In our experiments we further discovered the preconditions for pit enlargement. When the debris flow front collides with sediment, it immediately scours out the sediment, and the debris flow itself fills this scour volume in time. When it is difficult for the debris flow to penetrate the sediment rapidly, for example, in the case where the density of the debris flow is large and the particle size of the sediment is small, the debris flow will accumulate in the scouring pit. When a gathering debris flow cannot dissipate quickly, in this case, the flow direction of the subsequent debris flow will not change, and the scouring effect will also be weakened after the sediment surface is replaced by debris flow. Conversely, subsequent debris flow can continuously expand the pit, but the velocity of the debris flow out of the pit is significantly reduced due to the change in flow direction, resulting in a much lesser scouring effect after the pit; and finally, the gully bed presents the shape of a pit at the entrance and a groove in the middle and rear.

Through theoretical analysis, Pan^19^ and You^18^ proposed a critical a critical slope at which debris flow neither erodes nor deposits, and verified the accuracy of their method through experiments. However, due to various conditions, scouring or depositing is bound to occur in experiments. The method of how to determine the critical slope experimentally is not given further. In this paper, the critical scour slope range for the scour occurrence can be effectively determined based on the scour shape of the gully bed, and the effect of debris flow density and grain size of sediment on the critical scour slope is found. It should be noted that the scouring intensity of a debris flow is related to its velocity and depth. The flow velocity and flow depth in the erodible bed section will vary between flume models, which means that the final critical scour slope will also vary. However, this does not change the effect of changes in debris flow density and sediment grain size on the critical scour slope.

For the narrow-steep gully type of debris flow, most studies are still at the stage of field investigation and statistical analysis^9,30^. In the experiments, scouring occurred in narrow-steep gullies when the debris flow density is small, e.g., 1.7 g/cm^3^. The lower the debris flow density, and slope of the narrow-steep gully is high, the higher the debris flow velocity and therefore the easier it is for the debris flow to scour the gully bed. Once scouring has occurred, the scour pit will continue to expand as the small density of debris flow does not easily deposit on the pit. Sediment stability is lower on steeper slopes and as the scour pits continue to expand, sediment will continue to flow into the scour pit. When the slope is steep enough, there may even be a sudden collapse of a large amount of sediment, resulting in severe headward erosion.

There are still some shortcomings in the experiment. The grain size of the bed sediment in this experiment is relatively uniform, while that in the field always has a wider range, implying differences in porosity and granular viscosity between the field and the experiment^8,31^. These are important influencing factors and should be further considered in future experiments. We should also focus on more similar conditions than just geometric similarity.

## Conclusion

47 groups of flume model experiments were designed to investigate the erodible bed scoured by debris flow in the narrow-steep gully. The experimental work consists of capturing the geometric features of the erodible beds based on 3D laser scanning technology and recording the scouring process based on a high-speed camera by varying several parameters, including debris flow density (*ρ*), flume slope (*θ*) and grain size of the bed sediment (*D*). And the bed model is further processed to obtain scour shape, critical scour slope (*θ’*) and the maximum scouring depth (*H*_*m*_). The following main conclusions can be drawn:

- The overall characteristic of the scour shape is a pit at the entrance and a groove in the middle and rear, and the depth of the pit is much greater than the groove. With the enhancement of scouring, the position of the maximum scouring depth will tend to move downward. In times of particularly severe scouring, even headward erosion occurs.
- Based on the scour shape, the critical slope at which scour occurs exactly on the gully bed is determined. The critical scour slope is positive with debris flow density, but negative with the grain size of the bed sediment.
- As the debris flow density decreases, the maximum scouring depth increases correspondingly, and it also increases with the increasing grain size of the bed sediment and flume slope. The influence of the three variables mentioned above on the scour strength is further analyzed.
- For narrow-steep gullies, when the debris flow density is low, an excessive debris flow velocity occurs, resulting in the debris flow easily scouring the gully bed sediments; and due to the low stability of the sediments in narrow-steep gullies, the sediments near the scour pits are prone to collapse and even to headward erosion.

### Supplementary Information

Supplementary Figure S1.

## Data Availability

The datasets used and/or analyzed during the current study available from the corresponding author on reasonable request.
