# Supplementary material for: Experimental study of erodible bed scoured by the debris flow in the narrow-steep gully
Source: Sci Rep. 2023 Sep 9;13:14894. doi: 10.1038/s41598-023-41589-1 (PMC10492852; doi:10.1038/s41598-023-41589-1)
Supplement: Supplementary file 1 — Supplementary Figure S1. [file 41598_2023_41589_MOESM1_ESM.docx]

# Experimental study of erodible bed scoured by the debris flow in the narrow-steep gully

Yu Wu ^2^, Jiejie Ji ^3^, Shunchao Qi ^1,2^, Xiekang Wang ^1,2^, Dong Li ^2^, Hongtao Li ^1,2^, Xingguo Yang^1,2^ and Qiang Yao ^1,2,^*

^1^ State Key Laboratory of Hydraulics and Mountain River Engineering, Sichuan University, Chengdu, Sichuan 610065, China

^2^ College of Water Resource and Hydropower, Sichuan University, Chengdu, Sichuan 610065, China

^3^ Sichuan Water Resources and Hydroelectric Investigation, Design and Research Institute Co., Ltd, Chengdu, Sichuan 610021, China

* Correspondence: yaoqiang777@126.com


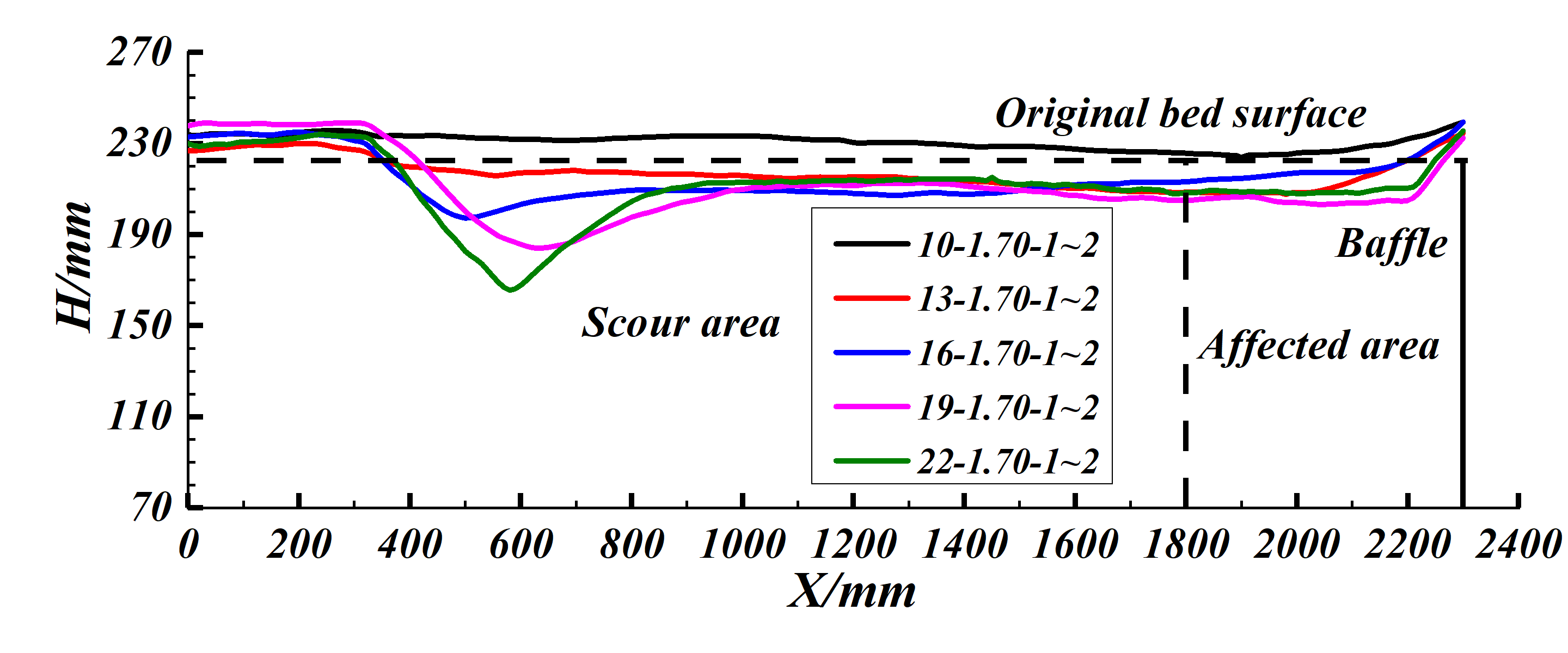


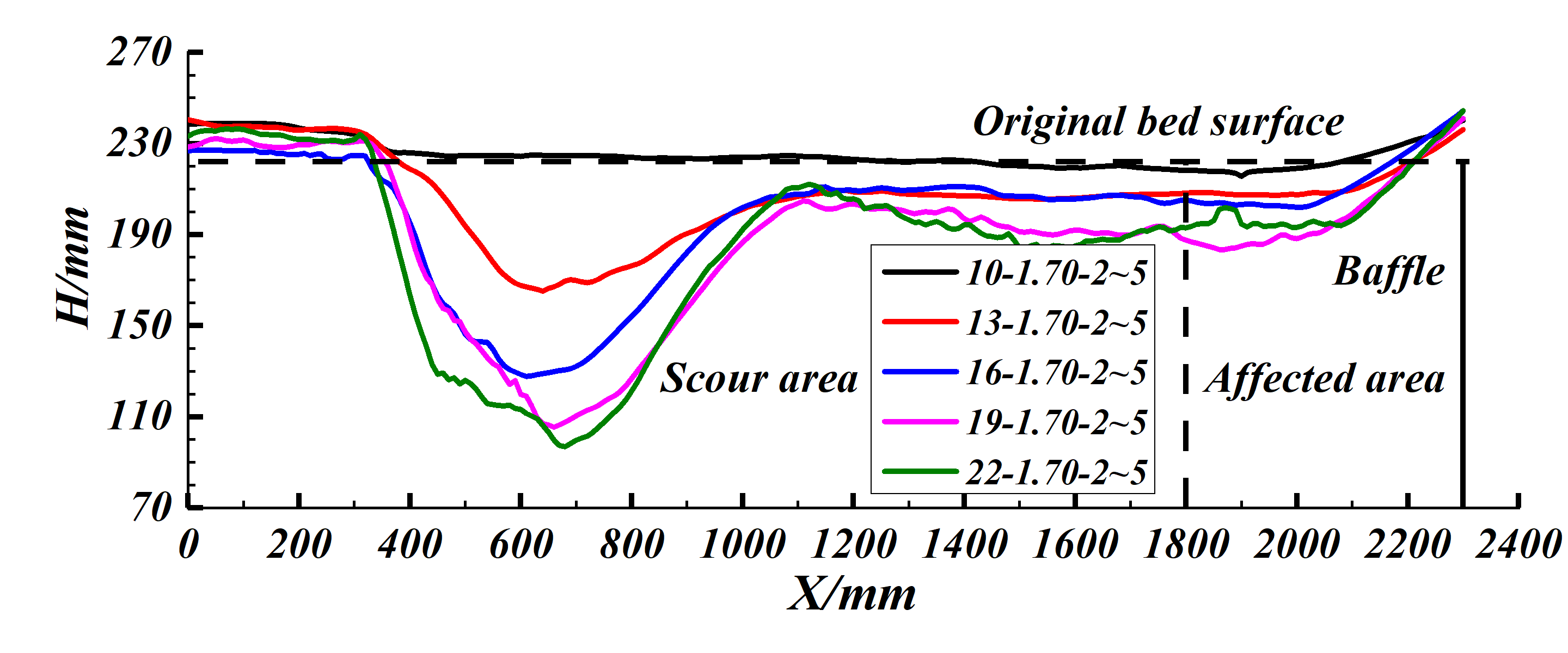

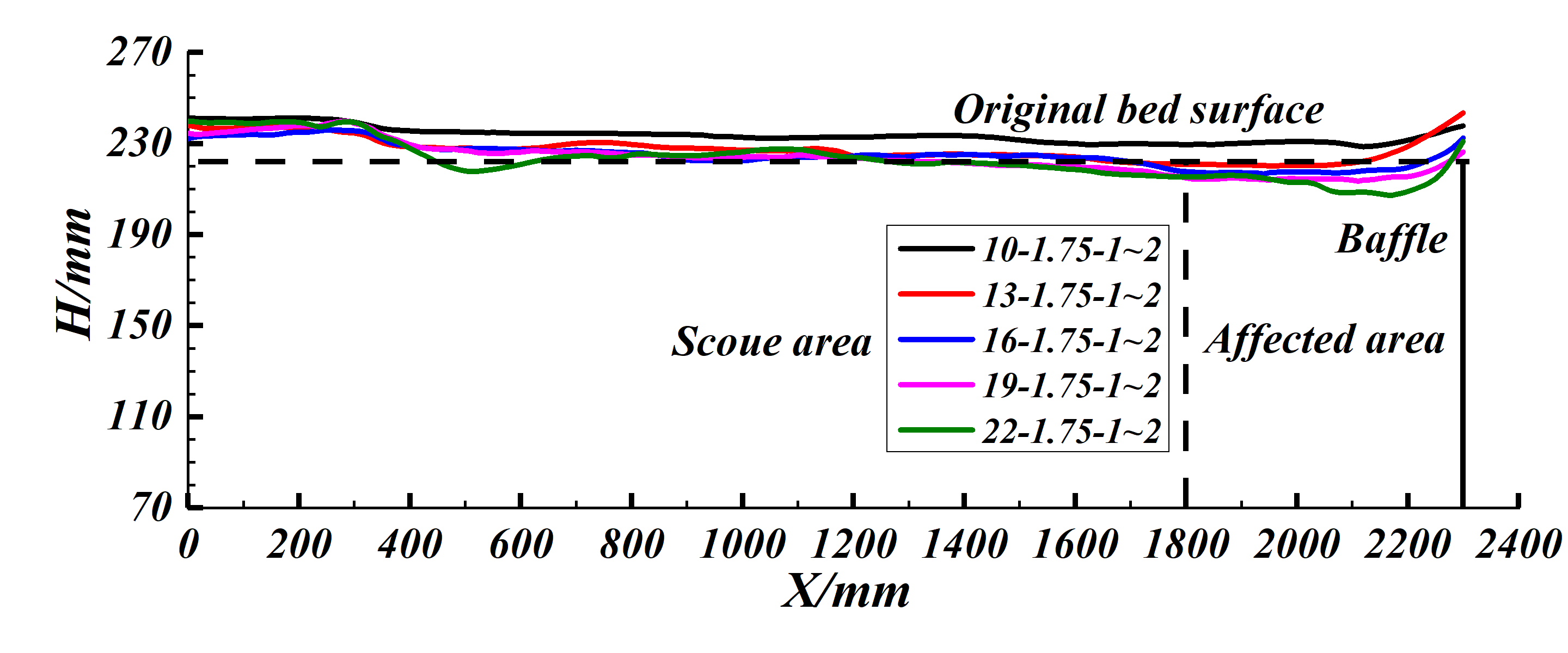

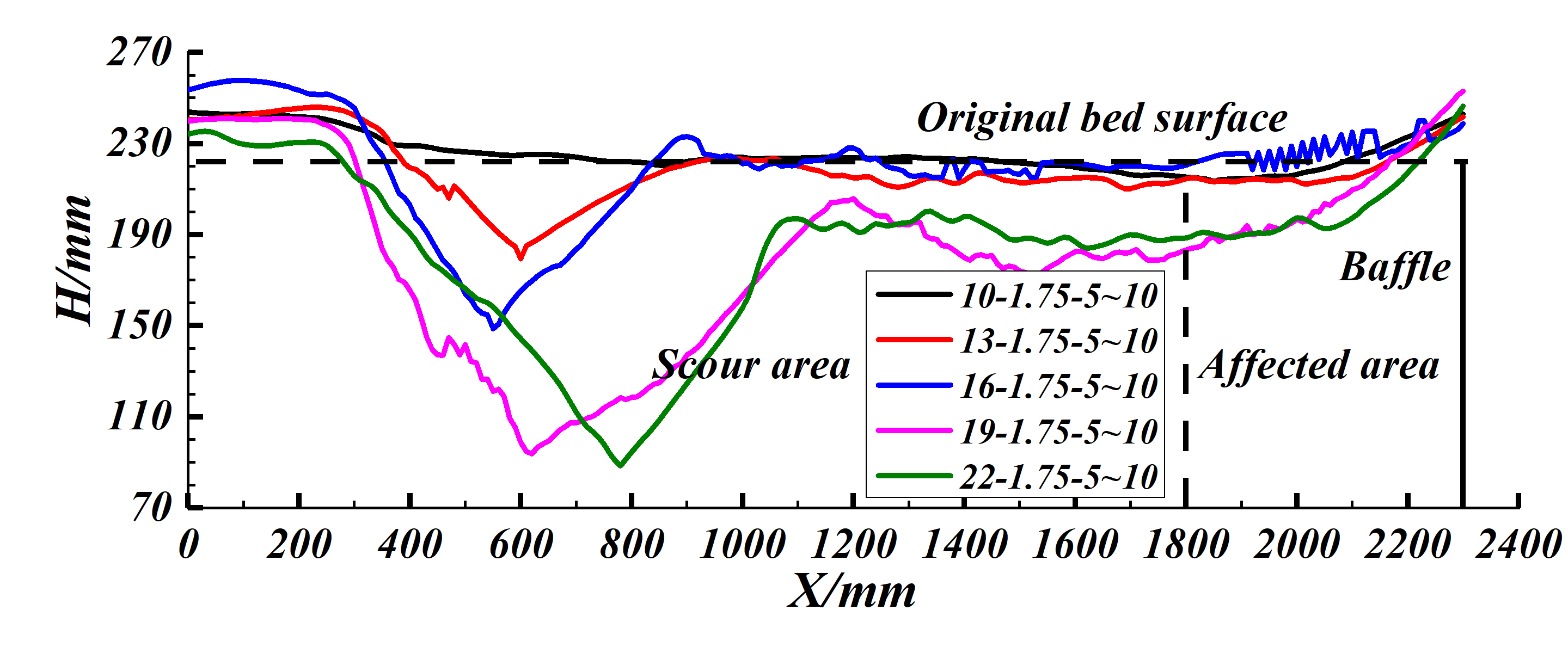

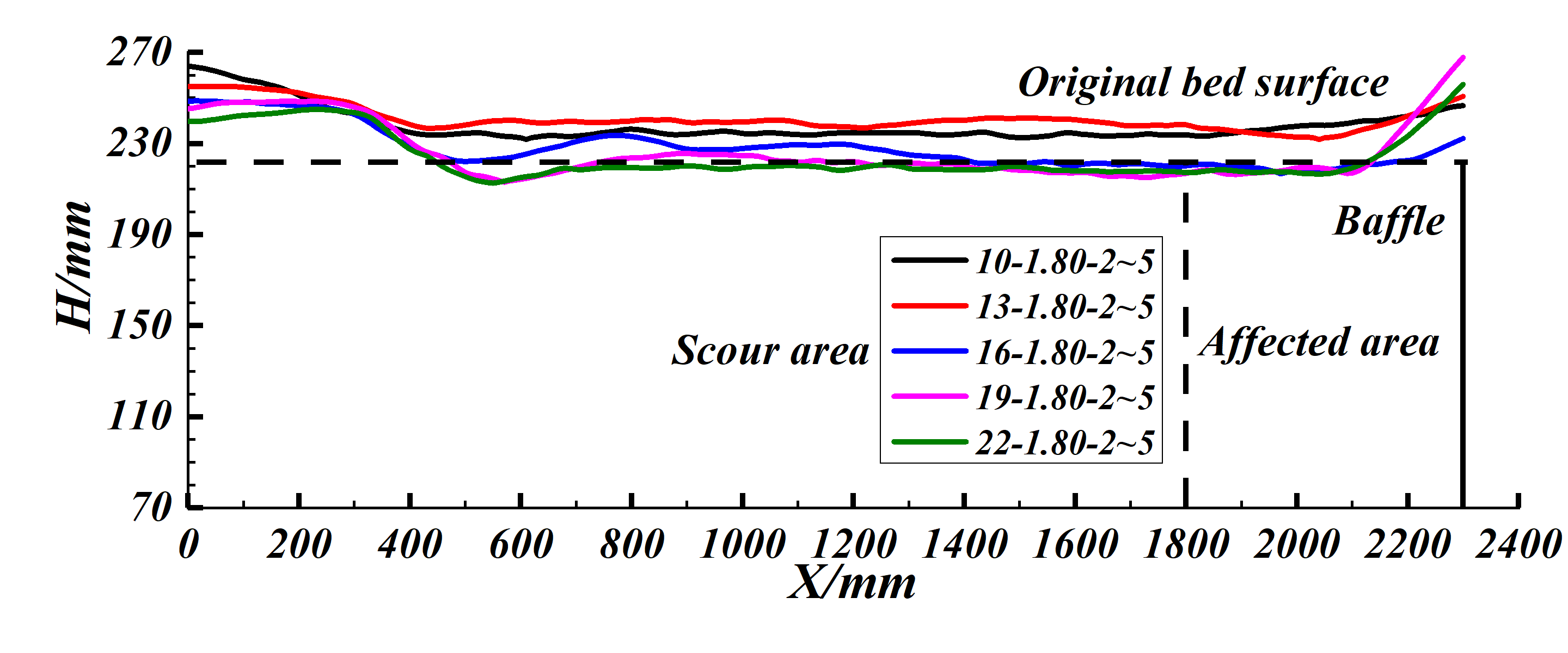

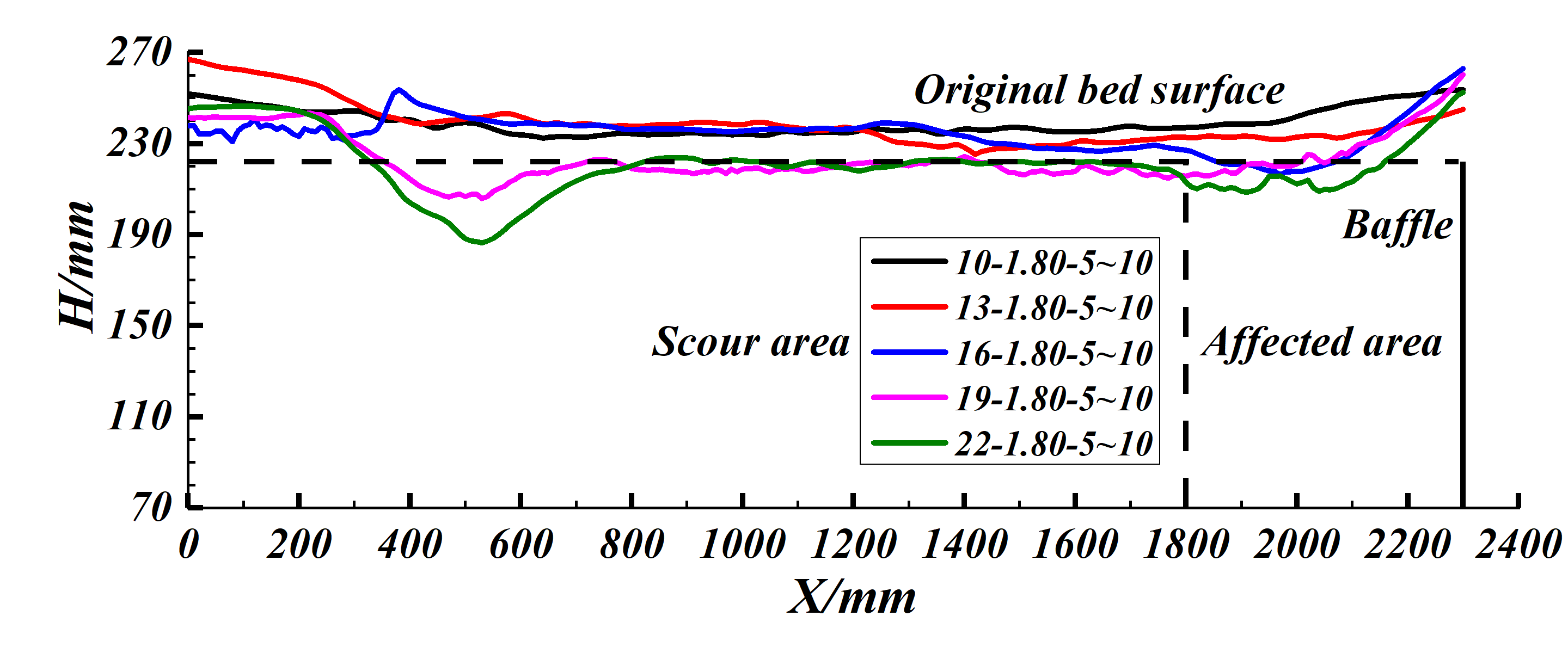


**Supplementary Figure S1** Longitudinal section of the erodible bed at the maximum scouring depth (where *H* is the sediment height; where *X* is the distance to the entrance of the erodible bed).
